# Evaluating artificial intelligence in medicine: phases of clinical research

**DOI:** 10.1093/jamiaopen/ooaa033

**Published:** 2020-09-08

**Authors:** Yoonyoung Park, Gretchen Purcell Jackson, Morgan A Foreman, Daniel Gruen, Jianying Hu, Amar K Das

**Affiliations:** o1 Center for Computational Health, IBM Research Cambridge, Cambridge, Massachusetts, USA; o2 Center for AI, Research, and Evaluation, IBM Watson Health, Cambridge, MA, USA; o3 Department of Pediatric Surgery, Vanderbilt University Medical Center, Nashville, Tennessee, USA; o4 Center for Computational Health, IBM T.J. Watson Research Center, Yorktown Heights, New York, USA

**Keywords:** AI (artificial intelligence), machine learning, health information technology, evaluation research

## Abstract

Increased scrutiny of artificial intelligence (AI) applications in healthcare highlights the need for real-world evaluations for effectiveness and unintended consequences. The complexity of healthcare, compounded by the user- and context-dependent nature of AI applications, calls for a multifaceted approach beyond traditional in silico evaluation of AI. We propose an interdisciplinary, phased research framework for evaluation of AI implementations in healthcare. We draw analogies to and highlight differences from the clinical trial phases for drugs and medical devices, and we present study design and methodological guidance for each stage.

## INTRODUCTION

The history of artificial intelligence (AI) dates back to the 1950s when Alan Turing introduced the idea of computers performing intelligent tasks.^1^ The term was coined by John McCarthy in 1956 at a Dartmouth conference.^2^ Twenty years later, the field entered the “AI Winter,” a consequence of optimistic promises and failures to keep them.^3^ As AI makes possible applications that can learn, adapt, and predict, blooming interest in AI offers the promise of an “AI Spring.”^4–6^ However, recent events also reveal unexpected risks of AI. A chatbot turned racist after training by online users, a racially biased tool used to inform parole decisions, and a fatal accident caused by an autonomous driving car are examples of unforeseen and severe consequences of AI.

One of the most fertile fields for application of AI is healthcare, enabled by vast data in electronic health records (EHRs) and enhanced computational power.^7–11^ AI applications offer enormous potential to improve patient care, from identifying new drug targets^12^ to supporting clinical decision making^13^ and lifestyle changes for disease prevention.^14^ Numerous *in silico* studies have assessed the accuracy of AI model predictions or concordance between human experts and algorithms.^15–17^ However, many clinicians and policy makers have criticized these foundational studies because the benefits and risks of AI have yet to be adequately measured in clinical practice.^18^ Such criticisms indicate a gap in the literature on the steps needed for a comprehensive evaluation of AI based on risks and benefits. In this article, we describe a phased approach for AI evaluation in healthcare, leveraging both prior informatics evaluation approaches and clinical trial phases required for approval of drugs and medical devices. We elaborate the research activities unique to AI in healthcare and draw parallels to the regulatory framework—a comparison frequently drawn in isolated examples, but not comprehensively articulated in the published literature.

### AI versus AI implementation in healthcare

Although definitions vary, AI is often characterized as sophisticated mathematical models employing techniques (such as deep learning) for learning and problem solving. Such tools can be assessed by statistical criteria quantified with computational experiments. In contrast, the implementation of AI in healthcare is *AI-based software that informs or influences clinical or administrative decisions and can affect health or healthcare delivery*. To determine the effects of AI tools in healthcare settings, comprehensive evaluation beyond computational performance is required. The evaluation focused only on the technical aspects of AI neglects the challenges of using AI in clinical practice, in which predictability, repeatability, explainability, and transparency are paramount.^19–22^

The high-stakes, regulated domain of medical drugs and devices can inform a structure for evaluation of AI solutions with a shared goal of ensuring the safety and effectiveness of health interventions. Table 1 shows a simplified view of the evaluation process for drugs and devices in the United States. Drug ingredients undergo quality control during preclinical development. After finding optimal dosage, toxicities, and evidence of efficacy in phases 1 and 2 trials, a large-scale phase 3 trial is conducted to assess therapeutic efficacy. Medical devices are evaluated through a cycle of development, proof-of-concept tests, quality improvement, and trials. For both, continuous surveillance is required to detect unexpected safety issues.^23^^,^^24^

**Table 1.** Evaluation for AI software compared to the approval processes of drug and devices for healthcare

Study phases	Drug	Device	AI in healthcare	Examples of study methods
Phase 0 Discovery and invention	Compound development In vitro/animal tests	User needs and workflow assessment Prototype design and development	User needs and workflow assessment Data quality check Algorithm development and performance evaluation Prototype design	Ethnographic studies to identify user needs, laboratory studies on limited data sets to measure algorithm prediction accuracy
Phase 1 Safety and dosage	Determine optimal dose Identify potential toxicities	Quality control Design updates	In silico algorithm performance optimization Usability tests	Determination of thresholds to balance sensitivity and specificity for a particular clinical use case, scenario-based testing to assess cognitive overload
Phase 2 Efficacy and side effects	Early efficacy tests Adverse event identification	Proof-of-concept tests Potential harm identification Design and quality improvement	Controlled algorithm performance/efficacy evaluation by intended users in medical setting Interface design Quality improvement	Retraining and reassessing model performance with larger real-world data sets, measurement of the efficiency of information delivery and workflow integration with representative users, pilot study of predictive algorithm in a clinical setting
Phase 3 Therapeutic efficacy	Clinical trial Adverse event identification	Clinical trial Adverse event identification	Clinical trial Adverse events identification	Randomized controlled trial to test whether delivery AI-based decision support affects clinical outcomes and/or results in user overtrust
Phase 4 Safety and effectiveness	Postmarketing surveillance	Postapproval studies	Postdeployment surveillance	Measurement of algorithmic performance drift

AI solutions for healthcare differ from drugs or medical devices in that they are designed to affect human decision making. The utility of conveyed information is determined by perception, comprehension, and subsequent actions of the user. Hence, assessing the effects of AI in medicine cannot be done independently from its intended users. Guidelines from the Food and Drug Administration on the regulation of machine-learning or AI-based applications contain high-level directions, but not specific guidance, on how to conduct each step of evaluation.^25–27^ We describe a framework for evaluation of AI in healthcare and methodological considerations for each phase (Table 2). This framework can help ensure that the quality of AI interventions meets expectations. Suggested methods derive from classic evaluation stages in the field of biomedical informatics but have not been clearly articulated as phases of clinical trials and research.^28–31^

**Table 2.** Study designs and considerations for each phase of evaluation of AI in healthcare

Study	Methods	Study phase	Study objectives	Considerations
Data quality control	Descriptive analyses	Phase 0	Ensure data quality meets certain standards and scope of data is relevant for the target population	Data quality and scope can change while numerous analytic choices are implemented
Algorithm testing	Statistical analysis	Phase 0	Evaluate AI algorithms for predictive accuracy or other performance metrics	The acceptable standard for accuracy depends on the clinical consequences of being incorrect across types of errors Separate training and test datasets avoid overfitting It may be necessary to establish baseline human performance of task being replicated
Ethnographic research	Observations Workflow analysis	Phase 0, 1	Identify user needs and understand workflow Determine useful functionalities and design options Understand social and cultural background which affect clinical decision making	Process is human resource intensive Studies may not generalize across settings This stage of activities should precede prototype development
Usability testing	Simulation	Phase 1, 2	Observe user activity in close-to-real scenarios to understand why something does and doesn’t work	Simulation cannot estimate the effect in the real-world scenarios Potential impact of artifacts from simulated scenarios
	A/B testing	Phase 1, 2	Conduct controlled experiment to provide an effect size estimate Test efficiently using crossover design	Controlled experiment environments are needed Carryover effect can invalidate crossover study results
	Experts’ review	Phase 1, 2	Consider usability testing by a small number of experts when budget and time is limited or direct reach to end user is difficult	Experts should take full context of use into account, such as clinical workflow and clinician mental models
Clinical trial	Individual randomized trial	Phase 3, 4	Can be blinded depending on the nature of implementation	Most robust study design are employed Without blinding, there’s a risk of contamination between proximal patients
	Cluster trial (parallel)	Phase 3, 4	Evaluate the cluster effect on a group of users or a healthcare facility	Possibly better than individual randomization due to AI affecting a group or cluster at a time Cluster size should be large enough for inference Potential effect of time-varying factors Should adjust for clustering effects in analyses
	Stepped-wedge trial	Phase 3, 4	Evaluate in real-world settings with staged introduction of implementation to multiple sites Get time-adjusted effect estimates	All sites receive implementation—desirable if implementation is thought to be beneficial This design is potentially more resource intensive as all sites receive implementation
	Pre–post comparison	Phase 3, 4	Evaluate using preimplementation data as its own control for postimplementation data of the same cohort	This design controls for time invariant factors, but can be subject to bias due to time-varying factors or underlying disease trends
Observational cohort study	Prospective cohort study	Phase 3, 4	Evaluate for effectiveness in nonrandomized, prospective cohorts	This design is less resource intensive than randomized trials and a better control for study design components than retrospective cohort studies Confounding bias is possible
	Retrospective cohort study	Phase 3, 4	Evaluate for effectiveness with fewer resources compared to trials or prospective studies by utilizing existing electronic health records or healthcare claims database	Data are not collected for research purpose, so requires knowledge of data generation and collection process, nature of missing data, etc. Potentially more prone to biases
User feedback	Feedback on product	All study phases	Understand any changes in user need related to the developing/developed product Assess satisfaction and perceived value of the users over time	A system should be built with a functionality to continuously collect user feedback and update based on the information
Continuous monitoring	Surveillance with data collection	All study phases	Monitor for unexpected adverse effect and adherence to the system	

### Phase 0: Discovery and invention

Phase 0 evaluation contains two parallel efforts: assessment of user needs and development of AI algorithms. Similar to devices, prototypes are developed prior to first in-human studies. Thus, activities such as identifying target users, understanding workflow, ensuring interpretability, and prototyping an initial design should begin in phase 0 and continue into subsequent phases. Explainability and user needs can be probed and assessed through an algorithm-informed question-bank approach for user-centered explainable AI design.^32^ Data quality checks must precede any other activity as the main “ingredient” of AI. For example, researchers should examine their data for validity (erroneous input), completeness (pattern and extent of missing data), biases (representativeness of the data), and timeliness (data reflecting current practice). Open-source toolkits, such as Aequitas^33^ or AI Fairness 360,^34^ can be used to evaluate metrics of bias and fairness in AI algorithms. Statistical performance metrics can then follow as criteria for further evaluation. Measurement of human performance is important to establish a baseline from which the accuracy of AI solution replicating human task can be judged.

### Phase 1: Technical performance and safety

Phase 1 involves finding a balance between the benefits and side effects of an intervention. For drugs, this phase determines the optimal dosage and identifies toxicities. For AI algorithms, phase I optimizes model performance for the application setting, such as determining a tradeoff between precision and recall. This task often requires domain knowledge to understand the clinical consequences of false positives or false negatives. If models were developed using previously collected data, Phase 1 is when real-world data evaluation should occur. Like toxic drugs, AI models may produce harmful results if algorithms are biased or based on incorrect information.^35^^,^^36^ Even with valid model outputs, design of AI solutions can lead to misperception or misunderstanding by users. The extent to which the AI models are understood by users can be a checkpoint for potential harm. In addition, what is deemed “intelligent” or “useful” can differ among users, unlike drugs or devices that have more clearly defined physical properties. Optimizing implementation of an AI model involves finding the most effective amount of information to provide to users, how and when to deliver it, and how to convey the model’s confidence in its insights. Such adjustments reflect the complexity of delivery of AI solutions compared to drugs, as the information provided may need to vary across different users.

Ethnography and applied social science methods from phase 0 can be used for understanding the interactions between users and AI solutions. Usability evaluation includes ensuring users are able to discover, understand, and use system features. Usability testing such as simulation studies or scenario-based testing that impose hypothetical clinical scenarios and ask the study participants to perform certain tasks can be employed to detect a cognitive overload or overtrust issues. Equally important is identifying potential risks, including workflow disruption, patient safety concerns, or model outputs that contradict clinician insights. Evaluation should also assess the extent to which mechanisms exist for catching and correcting errors including poor model fit, numerical instability, software malfunction, hardware malfunction, or human error.

### Phase 2: Efficacy and side effects

While the mechanism of actions for drugs or physical effects of devices are known by phase 2 trials, the ways in which AI solutions affect users and outcomes of interest may differ from expectations. Both unintended consequences and unintended benefits may be realized. Study participants’ activities and thought processes should be probed, externalized, and recorded to understand where and how the intended efficacy is achieved. AI algorithms are dynamic and often involve randomness during the course of insight generation. If users do not trust AI algorithms, solutions will most likely be undervalued. On the other hand, unforeseen adverse events may involve overreliance of decision makers on generated insights despite inherent statistical inaccuracies of AI models. Study designs such as A/B testing can evaluate relative efficacy and uncover unintended consequences.^37^ Most usability testing is done in laboratory settings, so what is measured in this phase is often an intermediate outcome for the desired outcome. For example, decisions more concordant with treatment guidelines would be expected to improve patient outcomes, and reduced time spent in administrative tasks would likely decrease costs. Validating improvement of intermediate measures is a critical step to justify larger phase 3 trials and to estimate their sample sizes.

### Phase 3: Therapeutic efficacy

The ultimate value of AI solutions in medicine is determined by clinical studies that assess whether they can improve health outcomes in real-world settings. The goal of phase 3 evaluation is to demonstrate efficacy and safety compared to the standard of care through well-designed, large-scale studies. In many cases, AI tools in healthcare work to enhance user’s performance, not to replace humans. Therefore, the comparison should be made between the performance of decision makers with and without AI tools, not the performance of decision makers versus AI models alone.

Clinical studies of drugs and devices are highly resource intensive and often require multiple sites, where dedicated personnel must gather data efficiently and track subjects reliably. Large-scale trials are undertaken by contract research organizations or clinical trial networks whose operations are separate from the health system. This approach may not work for AI solutions, as the data needed to create actionable information will be part of clinical practice, and the delivery system must be embedded into the clinical workflow. Timely evaluation requires research infrastructure to efficiently store and process collected data such as that in an EHR.

### Phase 4: Safety and effectiveness

Self-learning and self-improving capabilities throughout the lifecycle are distinct features of AI tools. As underlying data and software components can change and evolve over time, processes are required to ensure that the validity and quality of AI software are not compromised, and adverse effects do not arise from these changes. For example, the patient population affected by software may shift toward disease groups for which it was not originally intended. Just as antibiotic performance can be altered by emerging resistance, AI must be re-evaluated for efficacy and safety over time.

Valid causal inference from observational data requires careful adjustment for potential biases. There are numerous epidemiological and machine-learning methods that can be employed to account for confounding.^38–41^ In many cases, data collection can be automated through EHR systems, resulting in a passively accrued dataset reflecting outcomes and use. The efficiency of information delivery and integration into the medical workflow can be examined using such data. Additionally, AI applications can be instrumented to collect information about how specific features are used in practice, supporting the evaluation of their effect on outcomes. This large-scale, continuous collection of data is fundamental to learn and improve AI dynamically over time, but it can be complicated by the data security and privacy issues. Further effort is necessary to ensure that sensitive patient data are collected, stored, and used in appropriate ways.

## CONCLUSION

Deploying AI in healthcare is a high-stakes, high-reward endeavor. This manuscript proposes a comprehensive framework for the evaluation of AI solutions in healthcare. As for drugs and medical devices, research on AI requires sequential, long-term, and rigorous studies to generate scientifically valid evidence that is reproducible over time and across populations. In contrast to drugs and devices, the performance of AI tools in medicine depends on the understanding, trust, and subsequent behaviors of users. AI evaluation also requires integration into the existing clinical environment and a platform to collect, store, and process data, and to deliver the outputs to users in a timely manner. The comparison to the evaluation of drug and medical devices may facilitate an understanding of the evaluation of AI for the clinical audience, but the framework has limitations, especially for adaptive AI systems. For example, changes in underlying data and model performance from learning may necessitate concurrent revaluation of multiple phases. A comprehensive evaluation of AI tools across phases of research will require multidisciplinary teams with expertise in computer science, healthcare disciplines, and the social sciences. To minimize potential biases, ideally, developers should not evaluate their own tools, especially in the later phases of evaluation. Creation of collaborations across academic, public, and private institutions or dedicated evaluation teams without responsibility for solution development or sales may be necessary. Although certain AI tools in healthcare may be regulated, a commitment to the systematic evaluation should be an ethical responsibility of informatics professionals. Investing in the time, expertise, and resources needed to conduct studies of AI may ensure that patients and healthcare systems receive the promised benefits and enjoy a long “AI Summer” from these advancements.

## Authors’ contribution

Y.P. and A.D. conceived of the presented idea. All authors contributed to the design and refinement of the framework. 

## FUNDING

This research did not receive any specific grant from funding agencies in the public, commercial, or not-for-profit sectors.

## Conflict of interest statement

Y.P., G.P.J., M.F., D.G., J.H., and A.D. are employees of IBM. 
